# Effectiveness of psychosocial interventions to improve the mental health in men who have sex with men: a systematic review and meta-analysis

**DOI:** 10.3389/fpsyt.2025.1612755

**Published:** 2025-09-22

**Authors:** Shuochi Wei, Samantha Jeannie Cheng, Jiaxin Li, Edmond Pui Hang Choi, William Chi Wai Wong

**Affiliations:** ^1^ Department of Family Medicine and Primary Care, School of Clinical Medicine, Li Ka Shing Faculty of Medicine, The University of Hong Kong, Hong Kong, Hong Kong SAR, China; ^2^ Institute of Psychology, The Chinese Academy of Sciences, Beijing, China; ^3^ School of Nursing, Li Ka Shing Faculty of Medicine, The University of Hong Kong, Hong Kong, Hong Kong SAR, China; ^4^ Department of Family Medicine and Primary Care, The University of Hong Kong-Shenzhen Hospital, Shenzhen, China

**Keywords:** mental health, men who have sex with men, psychosocial intervention, review, meta-analysis

## Abstract

**Background:**

Men who have sex with men (MSM) are vulnerable to mental health problems. Some psychosocial interventions showed different effects on various mental health aspects, but the exact pooled effect size was uncertain. This study aimed to evaluate the effectiveness of psychosocial interventions for mental health among MSM.

**Methods:**

We included randomized controlled trials and quasi-experimental studies of psychosocial interventions aimed at improving the mental health of MSM. The outcomes were the effect sizes of overall mental health and specific aspects (depressive symptoms, anxiety symptoms, substance abuse, suicidal ideation, stress, coping, emotion, social function and identity). The fixed-effect or random-effect model was adopted to calculate the effect sizes. The study was registered with PROSPERO (CRD42024551392).

**Results:**

We included 14 studies conducted between 2010 and 2024. The effect size of intervention for overall mental health status was 0.14 (95%CI: 0.08-0.21, n=14, I2 = 28.23%). The interventions had positive effects in depressive symptoms (Hedges’ g=0.25, 95%CI: 0.09-0.41), anxiety symptoms (Hedges’ g=0.20, 95%CI: 0.12-0.29), substance abuse (Hedges’ g=0.19, 95%CI: 0.10-0.28), stress (Hedges’ g=0.18, 95%CI: 0.03-0.33), coping (Hedges’ g=0.21, 95%CI: 0.06-0.36), emotion (Hedges’ g=0.16, 95%CI: 0.06-0.25), and identity (Hedges’ g=0.19, 95%CI: 0.07-0.30). There was no publication bias.

**Conclusions:**

Psychosocial interventions have a small-to-moderate effect on improving the mental health status of the MSM. Our study provides a comprehensive evaluation of the intervention effect, with estimations of overall mental health status and some specific aspects.

**Systematic review registration:**

https://www.crd.york.ac.uk/prospero/, identifier CRD42024551392.

## 1 Introduction

Mental health is a basic human right for all people which is crucial to personal, community, and socio-economic development (1). However, the discrimination and stigma based on sexual orientation impede the right of men who have sex with men (MSM) to the highest attainable standard of physical and mental health (2). MSM are more vulnerable to poor mental health as they face more stigma and stress in their daily lives (3). Despite significant progress in creating LGBTQ+-friendly societies and mental health promotions, MSM still reported disproportionate mental and psychological conditions like depression, anxiety, distress, negative feelings, and emotions, or low self-identification (4, 5). It is estimated that around 35% of MSM worldwide had experienced depressive symptoms, much higher than the general population (13%) (6). The pooled prevalence of depression among MSM was 37% in Asia, 26% in Europe, 34% in Africa, and 35% in the Americas (6). Another study estimated that 21% of MSM had suicidal ideation, with a pooled prevalence of 20% in Asia and 19% in the Americas (7). These regional differences both reflect widespread contextual issues highlighting regional needs and expectations. Meanwhile, inadequate mental health care for MSM could further exacerbate the mental state of MSM, leading to a poor quality of life (8).

Systemic factors critically shape MSM’s mental health through legal and policy environments. Criminalization of same-sex acts exacerbates stigma, limits health access, and increases psychological distress (9). Conversely, protective laws that reduce structural discrimination by ensuring access to healthcare, in particular mental health services, and legal recourse against discrimination, have shown to improve mental well-being of MSM population (9, 10). The variety of laws and policies across different countries underscores their impact on either exacerbating or alleviating mental health disparities, highlighting the need for context-specific interventions.

Psychosocial interventions refer to non-pharmacological interventions that focus on psychological or social factors, aimed to improve symptoms, functioning, quality of life, and social inclusion when used in people with mental health conditions (11). Psychosocial interventions are an essential component of mental health services and contribute to improving the mental health and well-being of MSM (12). The current interventions for MSM are mainly cognitive behavior therapy (13), supportive groups (14), education (15), online self-care (16), etc. Some studies showed a better intervention effect for MSM on depressive symptoms, distress, self-efficacy, and coping by education and other forms of interventions (12, 15). Previous reviews showed that psychosocial interventions could reduce depressive and anxiety symptoms in sexual and gender minorities and MSM, and decrease substance abuse in MSM (17, 18).

According to World Health Organization, as a state of well-being, mental health is more than the absence of mental disorders, it also includes emotional well-being, good behavioral adjustment, relative freedom from anxiety and disabling symptoms, and a capacity to establish constructive relationships and cope with the ordinary demands and stresses of life (19). From the global perspective, it is necessary to conduct a comprehensive evaluation of the overall effectiveness of psychosocial interventions and their components for MSM to assess the effectiveness and provide evidence for developing quality and affordable mental health care to improve the well-being of MSM.

This study aimed to conduct a systematic review and meta-analysis to evaluate the effectiveness of psychosocial interventions for mental health among MSM, with a particular focus on both overall effects and specific outcomes including depression symptoms, anxiety symptoms, substance abuse, suicidal ideation, stress, coping, emotion, social function and identity. This study would also compare the effects of interventions with different characteristics, such as face-to-face intervention and online intervention, self-care and interventions provided by professionals, etc. The hypothesis was that psychosocial interventions would have superior effects in improving overall mental health and specific outcomes compared to the control. This study would provide evidence-based recommendations for mental health care providers to better address the mental health challenges faced by MSM and improve their overall health and well-being. This study addresses key policy gaps in global mental health by evaluating psychosocial interventions for MSM. It demonstrates how interventions can reduce inequalities, safeguard the human rights of MSM, and inform reforms that align mental health services with global commitments to equality, non-discrimination, and equitable healthcare, thereby providing policymakers with evidence to improve services and reduce disparities.

## 2 Methods

### 2.1 Search strategy and selection criteria

In this systematic review and meta-analysis, we included studies based on the following criteria: (1) Participants were targeted at MSM or LGBTQ+ that included the MSM population; (2) The study was a randomized controlled trial (RCT) or quasi-experimental intervention study; (3) The psychosocial interventions were designed to improve the mental health of MSM or sexual minorities including MSM, in any form and of any frequency; (4) The outcomes were related to mental health symptoms or psychosocial function; and (5) Only studies published in English were included. We excluded studies if: (1) Its participants were MSM living with HIV; (2) The intervention was focused solely on the reduction the substance abuse amongst MSM; and (3) No access to the full original text or no access to contact the authors for full text and data.

The studies were searched from six sources, including four English databases (PubMed, PsycINFO, EmBase, and Cochrane Library) and two clinical registers (ClinicalTrials.gov and International Clinical Trials Registry Platform). The studies were searched on March 4, 2024, without a publish time limitation, by two researchers separately. The literature search strategies combined the keywords, Medical Subject Heading (MeSH) terms, and Boolean operators (AND, OR, NOT). The detailed search strategies were provided in the **Supplementary Materials (S2-7)**. Studies were screened by title, abstract, and full text. Literature was screened for eligibility by SCW and SJC. Each reviewer had a degree in psychology. Before screening and data extraction, the first author (SCW) developed a guideline to ensure consistency between reviewers. Disagreements were resolved by the third independent researcher (WW).

This study protocol was registered with PROSPERO (CRD42024551392) and followed the PRISMA reporting guidelines (S1) (20).

### 2.2 Data extraction and coding

Data extraction and data coding from each eligible publication were conducted by two reviewers (SCW and SJC) and overseen by a third independent person (WW) should any disputes arise. Reviewers extracted study information (author, year, title, study design, country), participant information (age, sample size, proportion of MSM), intervention setting (type, theory and framework, brief content, provider, number of sessions, duration, frequency, follow-up duration, delivery format, adherence), and outcome data (measurement scale, data at baseline and follow-up in intervention and control groups, adverse event). For data extraction of outcomes, the mean and standard deviation of continuous data will be extracted. If only standard errors were reported, the standard errors would be converted to standard deviations. If there were more than one comparison group, data were collected only from the group that received less intervention. If there were multiple follow-up time points, only the data from the last follow-up will be collected. The intervention types would be coded according to the adopted therapy; the delivery format as online intervention or face-to-face intervention; and the content as MSM-specific or for general LGBTQ+. The first reviewer drafted a data extraction and coding manual to ensure good interrater reliability. Two reviewers adopted an Excel form to conduct the data extraction and coding independently and checked the data together.

The Cochrane risk-of-bias tool for randomized trials (RoB-2) was adopted to evaluate the risk of bias in each included RCT study (21). According to RoB-2, the studies would be assessed as “low concerns”, “some concerns”, and “high concerns”. The Risk of Bias in Non-randomized Studies–of Interventions (ROBINS-I) was adopted to assess the risk of bias in quasi-experimental studies (22). Studies would be assessed as “low risk”, “moderate risk”, “serious risk”, and “critical risk”. The certainty of evidence would be assessed by Grading of Recommendations, Assessment, Development, and Evaluations (GRADE) (23). Two reviewers (SCW and SJC) conducted the assessment separately and reached an agreement after discussion. During the process of data extraction and coding, only a few disagreements were met and were solved quickly.

### 2.3 Data analysis

Considering that there were multiple different outcome measurements across studies, the standardized mean difference (SMD) was more appropriate for calculating the effects of each study (24). Hedges’ *g* was a statistical correction to the SMD and was adopted to estimate the effect size of the difference of changed scores from pre-intervention to post-intervention between the intervention group and comparison group in this study (24). The effect size would be considered as small (0·2), medium (0·5), and large (0·8) according to the Hedges’ *g* value. The heterogeneity between studies would be calculated by *I^2^* and Q statistics. The heterogeneity would be considered as not important (*I^2^*: 0-40%), moderate (30%-60%), substantial (50%-90%), and considerable (75%-100%) (25). A fixed model would be adopted if the *I^2^* was less than 50% or the *p*-value of the *Q* statistic was more than 0·05. A random model would be adopted if the *I^2^* was higher than 50% or the p-value of the *Q* statistic was less than 0·05. A positive Hedges’ *g* means a superior effect of the intervention. The funnel plot and Egger’s test were used to test the publication bias.

The primary outcome was the overall effect size of the intervention. The secondary outcomes were the effect sizes of the interventions on specific mental health and psychosocial function domains. We extracted all the specific interested outcomes from each study and coded them into the mental health domain and psychosocial function domain. The mental health domain included the measurements of depressive symptoms, anxiety symptoms, substance abuse symptoms, and suicide-related symptoms. The psychosocial function domain included the measurements of stress, coping, emotion, social function, and identity. First, we collected data on each specific outcome, like depressive symptoms and stress, to calculate the effect sizes of interventions on each outcome. Then, we would calculate two combined effect sizes of mental health and psychosocial function domains by aggregating the effect sizes of specific outcomes. The combined effect sizes were calculated by using the R package by Del Re et al. (26) We hypothesized that there was a high degree of correlation between the outcomes in the two domains and therefore we used 0.8 as the correlation coefficient in the aggregation. The overall effect size was calculated by combining the effect sizes of mental health and psychosocial function domains by using the same method. We used 0.6 as the moderate correlation coefficient when combining the two effect sizes. Such a statistical method was mainly based on one previous study (27). Sensitivity analysis would be conducted by excluding the studies with a high risk of bias.

Moderator effects would be assessed by subgroup analysis and meta-regression analysis. Subgroup analysis would be conducted according to different study characteristics, including the population (for MSM-only or sexual minorities including MSM), delivery formats (online or face-to-face), duration or session (low or high frequency), and intervention providers (self-care or by trained specialists). Meta-regression would assess the effect of continuous variables including the age of participants, and the number of sessions. The data analyses were conducted in Comprehensive Meta-analysis version 2 (CMA v2) and the calculation of data aggregation was conducted by the MAd package in R. There is no funding source for this study.

## 3 Results

**Figure 1:**
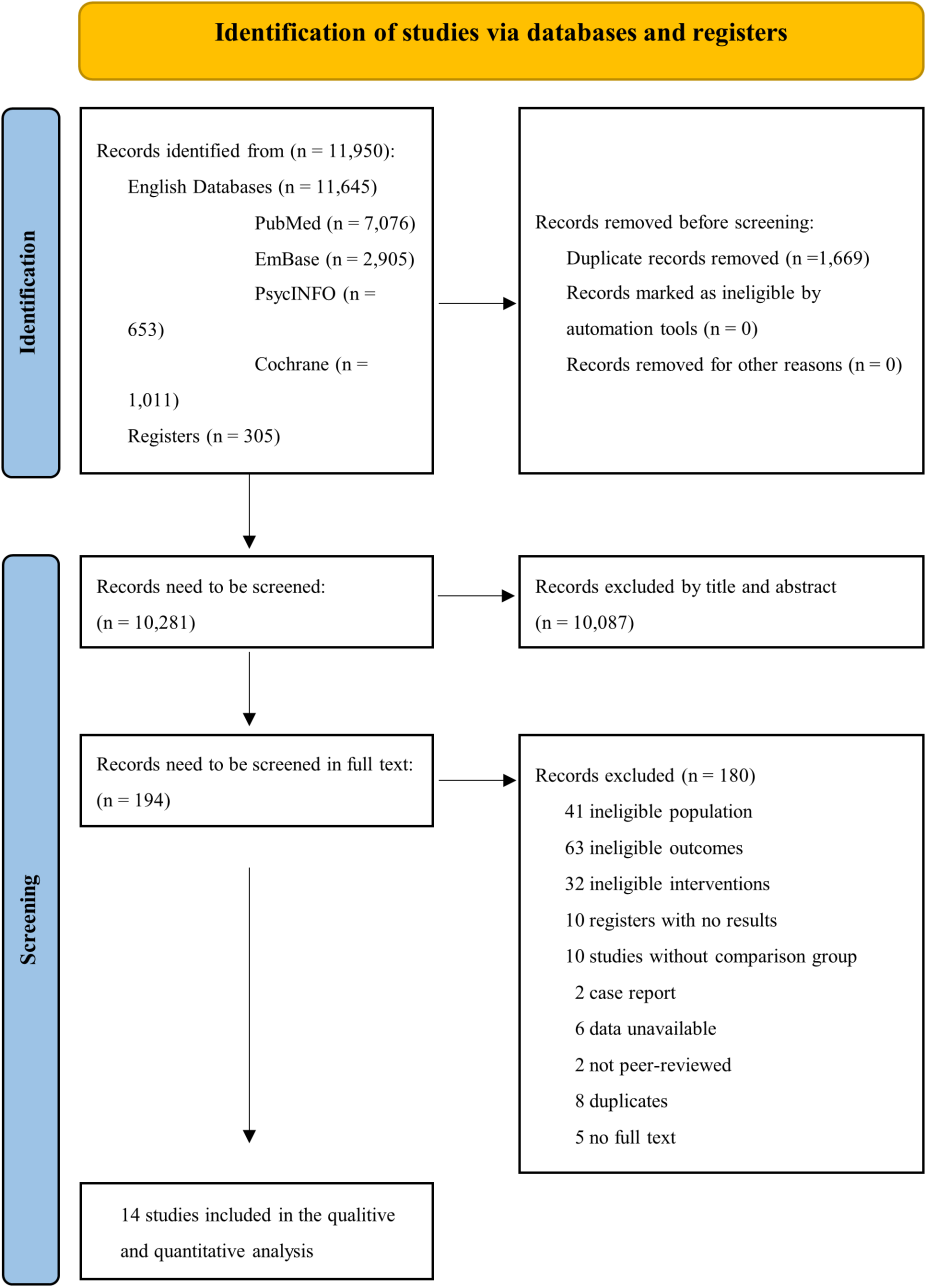
The literature screening process.

A total of 11,950 results were searched from four databases and two registers. 1,669 records were duplicates and 10,087 results were excluded by title and abstract screening. Fourteen studies met the inclusion criteria and were included in this study after the full-text screening of 194 studies (**Figure 1**) (13–16, 28–37).

There were 11 (78·57%) RCT studies and 3 (21·43%) quasi-experimental studies (**Table 1**). All the studies were published between 2010 and 2024, and most (78·57%) were published in the last five years. Eleven studies (78·57%) were conducted in the USA, one (7·14%) from China, one (7·14%) from Austria and Germany, and one (7·14%) from Canada.

**Table 1:** Characteristics of studies included in the systematic review.

Authors (year)	Study design	Participants			Intervention			Comparison	Outcome
		Participant	MSM ratio*	Age, M(SD)	Intervention type	Delivery	Details		
Beard et al (2017) (28)	Quasi-experimental	LGBQ and heterosexual individuals (84/357)	NI	34.42(13.46)	CBT+DBT	Group: up to five groups each day, five days per week; Individual: 2 to 3 sessions each week	Treatment comprised individual and group therapy focusing on the acquisition of CBT and DBT skills. Group content was derived from empirically supported behavioural manuals, including behavioural activation; identifying and challenging negative automatic thoughts; interpersonal effectiveness, and exposure therapy.	Heterosexual	BASIS-24; GAD-7; PHQ-9
Bauermeister et al (2022) (15)	RCT	Sexual and gender minority youth (135/135)	17.4%-53%	16.49(1.49)	Psychoeducation (online)	28 days, recommend at least twice a week	The application delivers fully automated information and skill practice across guides covering four content areas: (1) gender identity exploration, (2) sexual orientation and broader LGBTQ+ identity exploration, (3) stress and coping, and (4) internalized homophobia and transphobia. Each of the four guides comprises four types of content: (1) learning segments, (2) activities, (3) community content, and (4) external resources. Learning segments provide information about LGBTQ+ relevant vocabulary, queer history, and psychoeducation on minority stress and internalized stigma. Activities include interactive exercises. Community content includes video, audio, and written stories and images of LGBTQ+ youth. External resources connect youth to externally linked content designed for LGBTQ+ youth.	Control (general resource webpages without intervention content)	SAMA; COPE; LGBPIM; CDSI; TB; GAD-7; PHQ-8
Pachankis et al (2010) (29)	RCT	Gay male undergraduates (27/25)	100%	20.19(1.99)	Expressive writing (online)	3 sessions in 3 days	Expressive writing about gay-related stress; enhances exposure and eventual habituation.	Control (neutral topic)	SCL-90; CES-D; PANAS; GRRSS; RSES; Comfort; Openness
Pachankis et al (2022) (30)	RCT	Young gay and bisexual men (100/52)	99%	26.55(4.17)	CBT	10 sessions in 4 months	ESTEEM is a 10-session CBT intervention for reducing stress-sensitive mental health disorders by enhancing emotion regulation skills; reducing avoidance patterns, and improving motivation and self-efficacy for behaviour change. The Unified Protocol employs modules for motivation enhancement, interoceptive and situational exposure, cognitive restructuring, mindfulness, and self-monitoring techniques. Through an extensive adaptation process, the intervention adapted the Unified Protocol to enhance young gay and bisexual men’s stigma coping by reducing minority stress processes.	Control (HIV testing and counselling)	GRRSS; IHS; SOCS; DERS; Brooding; RAS; HAMD-17; ODSIS; BAI; OASIS; SIAS; BSI; SIDAS; AUDIT; SIP-AD
Pachankis et al (2015) (13)	RCT	Young adult gay and bisexual men (32/31)	100%	25.94(4.24)	CBT	10 sessions in 3 months	The intervention includes: enhancing emotion regulation abilities; reducing maladaptive cognitive, affective, and behavioural avoidance patterns; and improving motivation and self-efficacy for enacting behaviour change. Intervention modules are adapted to help participants identify minority stress experiences; track cognitive, affective, and behavioural reactions to minority stress, with a focus on avoidance reactions, including substance use and condomless anal sex; attribute distress to minority stress rather than to personal failure; and assertiveness training for coping with minority stress in safe situations.	Waiting list	CES-D; ODSIS; OASIS; AUDLT; TLFB; MOGS; GRRSS; IHP; SOCS; RRS; DERS; MSPSS; RAS
Pachankis et al (2020) (31)	RCT	LGBTQ young adult (36/36)	24.07%-51.85%	23.68 (3.11)	Expressive writing (online)	3 sessions in 3 days	Expressive writing prompts individuals to write about personally stressful events, potentially enabling cognitive processing of unresolved, psychological and physiological stressors.	Control (neutral topic)	CES-D; BSI; BAI; SIDAS; AUDIT; SIP-DU
Pachankis et al (2023) (32)	RCT	Sexual minority youth (60/60)	15.83%-50%	20.37(2.81)	CBT (online)	10 sessions in 4 months	The LGBTQ-affirmative ICBT sessions are based on the published client workbook and include: (1) setting goals and building motivation for LGBTQ-affirmative CBT; (2) monitoring LGBTQ-related stress and emotions; (3) understanding the nature and emotional impact of LGBTQ-related stress; (4) increasing mindful awareness of LGBTQ-related stress reactions; (5) increasing cognitive flexibility; (6) understanding emotion avoidance; (7) countering emotional behaviors; (8) experimenting with new reactions to LGBTQ-related stress; (9) emotion exposures for countering LGBTQ-related stress; and (10) recognizing accomplishments and looking to the future.	Control (assessment-only)	BSI; CES-D; ODSIS; OASIS; AUDIT
Poon et al (2022) (33)	Quasi-experimental	LGBQ adolescents and non-LGBQ peers (16/23)	NI	15.21(1.65)	DBT	18 sessions in 18 weeks	DBT- A is delivered with fidelity to the standard model including a weekly multifamily skills training group, individual therapy, 24/7 phone coaching, and a therapist consultation team.	Non-LGBQ peers	DERS; BDI-II; BAI; DBT-WCCL; BSL
Shen et al (2023) (16)	RCT	LGBTQ+ adolescents (262/276)	24.16%-48.51%	15.06(0.97)	CBT (online)	1 session (30 min)	Project RISE is a 20–30 min self-guided SSI explicitly targeting internalized stigma and minority stress reactions. RISE includes five general content sections: (1) an introduction to minority stress, privilege, and marginalization; (2) psychoeducation on the effects of minority stress; (3) stories from other youths about their experiences with minority stress; (4) interactive components wherein participants reflect on their identities and experiences with minority stress, identify related emotions and cognitions, and determine actionable, values-based needs; and (5) an exercise in which participants identify a coping statement to help them get through minority stress.	Active control	LGBIS- Identity Affirmation subscale; LGBIS- Internalized Homonegativity subscale; BHS-4; SHS; CDI 2:SR[S]; GAD-7
Goldbach et al (2021) (14)	RCT	LGBTQ adolescents (27/19)	18.2%-59.1%	15.14(1.28)	Psychoeducation	10 sessions in 10 weeks	The intervention relies on a mix of psychoeducation, didactic discussion, and interactive activities including (a) stress and coping; (b) disclosure decision-making; (c) family; (d) school-related stress and resilience; (e) peers and friendship; (f) safety in relationships; (g) spirituality, faith, and religion; (h) race, ethnicity, and social justice; (i) the LGBT community and history; and (j) intersections of health, substance use, HIV, and the medical system.	Control (as usual)	SMASI; BAI; PCL; BDI-II; CSSRS; PTSD 6-item
McDanal et al (2022) (34)	Quasi-experimental	LGBTQ+ youths (82/102)	NI	NI	Psychoeducation (online)	1 session (30 min)	Project Personality focuses on the malleability of traits and symptoms to strengthen adolescents’ perceived control and reduce hopelessness. Project CARE focuses on acting with self-compassion to systematically reduce self-hate, and Project ABC focuses on behavioural activation principles to demonstrate that engaging in valued activities can powerfully shape one’s mood.	Cisgender heterosexual	BHS-4; SHS; SHS†
Kirchner et al (2022) (35)	RCT	LGBTQ+ youths (242/241)	24.33%-51.87%	19.06(2.24)	Psychoeducation (online)	1 session (<10 min)	The project aims to empower youth by featuring personal video narratives of coping with difficulties during coming out.	Control (neutral)	RLIA; BHS-10; ASS; GHSQ; LGBIS
Craig et al (2021) (36)	RCT	LGBTQA+ Youth and Young Adults (46/50)	12.5%-27.08%	21.17 (4.52)	CBT (online)	8 sessions in 8 weeks	AFFIRM is a manualised affirmative CBT 8-session group. The curriculum includes an orientation (group norms, confidentiality, curriculum); sessions 1–2 (overview of CBT and the impact of minority stressors); sessions 3–4 (CBT, thought stopping); sessions 5–6 (coping skills, goal setting and cultivating hope); and sessions 7–8 (social support, self-compassion, self-care plans). Each session is sequenced to have: (a) a group check-in, review of previous sessions, and homework; (b) session objectives; (c) description, practice, and rehearsal of behavioural activities; and (d) reflective check-out and summary.	Waiting list	BDI-II; SAMA; BCI; RCS; HS
NCT04718194 (2024) (37)	RCT	Young Chinese MSM (60/60)	100%	23.22(3.26)	CBT (online)	10 sessions in 10 weeks	This online CBT treatment consists of 10 weekly modules. Modules contain weekly psychoeducational text and vignettes about minority stress and mental health; brief videos illustrating the CBT skills; and homework exercises that therapists review and provide feedback on. Homework exercises include weekly tracking of stress and mood, practicing new skills (e.g., mindfulness, cognitive restructuring), and exercises related to considering the origins of stress and negative emotions that participants may be experiencing. Therapists provide feedback after each homework assignment, including reviewing each participant's treatment goals as part of the first session's homework.	Control (self-monitoring)	PHQ-9; GAD-7; AUDIT; DUDIT; SIDAS; LGBIS; DERS-SF; MSPSS; RRS; ODSIS; OASIS; RSES

*The lower limit of the MSM ratio is the proportion of gay and the upper limit is the proportion of gay plus the proportion of bisexual. NI, No information; CBT, Cognitive behavioural therapy; DBT, Dialectical behaviour therapy; BASIS-24, The 24-item Behaviour and Symptom Identification Scale; GAD-7, The 7-item Generalized Anxiety Disorder Scale; PHQ-9, The 9-item Patient Health Questionnaire; RCT, Randomised Controlled Trials; SAMA, The Stress Appraisal Measure for Adolescents; COPE, Coping Orientation to Problems Experienced Inventory; LGBPIM, Lesbian, Gay, and Bisexual Positive Identity Measure; CDSI, The 5-item Coping with Discrimination Scale–Internalization; TB, Thwarted Belongingness subscale of the Interpersonal Needs Questionnaire; PHQ-8, The 8-item Patient Health Questionnaire; SCL-90, Symptom Checklist–90; CES-D, The Centre for Epidemiologic Studies Depression Scale; PANAS, Positive and Negative Affect Schedule; GRRSS, Gay-Related Rejection Sensitivity.

Scale; RSES, Rosenberg Self-Esteem Scale; HIS, Internalized Homonegativity Scale; SOCS, Sexual Orientation Concealment Scale; DERS, Difficulties of Emotion Regulation Scale; Brooding, Brooding Subscale of the Ruminative Response Scale; RAS, Rathus Assertiveness Schedule; HAMD-17, The 17-item Hamilton Depression Rating Scale; ODSIS, Overall Depression Severity & Impairment Scale; BAI, Beck Anxiety Inventory; OASIS, Overall Anxiety Severity & Impairment Scale; SIAS, Social Interaction Anxiety Scale; BSI, Brief Symptom Inventory; SIDAS, Suicidal Ideation Attributes Scale; AUDIT, Alcohol Use Disorders Identification Test; SIP-AD, Short Inventory of Problems–Alcohol and Drugs; TLFB, 90-day Time Line Follow Back; MOGS, Measure of Gay-Related Stress; RRS, Ruminative Responses Scale; MSPSS, Multidimensional Scale of Perceived Social Support; BDI-II, Beck Depression Inventory, 2nd edition; DBT-WCCL, The Dialectical Behaviour Therapy Ways of Coping Checklist; BSL, Borderline Symptoms List; LGBIS, Lesbian, Gay, and Bisexual Identity Scale; BHS-4, Beck Hopelessness Scale-4; SHS, Self-Hate Scale; CDI 2,SR[S], Children's Depression Inventory, Second Edition, Self-Report Short version; SMASI, Sexual Minority Adolescent Stress Inventory; PCL, The PTSD Checklist for DSM-5; CSSRS, Columbia Suicide Severity Rating Scale; SHS, State Hope Scale; SHS†, Self-Hate Scale; RLIA, Reasons for Living Inventory-Adolescents; BHS-10, Beck Hopelessness Scale-10; ASS, Affective State Scale; GHSQ, The General Help-Seeking Questionnaire; BCI, The Brief COPE Inventory; RCS, Proactive Coping Inventory for Adolescents-A (PCI-A)—Reflective Coping Subscale; HS, The 12-item Hope Scale; DERS-SF, The 18-item Difficulties in Emotion Regulation Scale-Short Form.

A total of 2,676 participants were recruited including 1,209 participants in intervention groups (median: 60, IQR^1^‡: 33-96) and 1,467 participants in comparison groups (median: 56, IQR: 32·25-126·75). The participants were primarily adolescents or young adults, with a mean age between 15 and 34 years old. Four studies (28·57%) only recruited MSM population or gay and bisexual men, and the rest recruited both MSM and other sexual minorities like lesbian and bisexual women with a proportion of MSM ranging from 12·1% to 59·1% (**Table 1**). The included studies measured various mental health and psychosocial function outcomes, mostly self-reported. The most frequently measured outcomes were symptoms of depression, which were evaluated in 11 of the studies and symptoms of anxiety, which were evaluated in nine studies. Substance abuse was assessed in six of them. Seven studies measured coping-related outcomes; eight on emotion-related outcomes; five on self-identity-related and social function-related outcomes, and five on stress-related outcomes (**Table 1**).

Among the 14 studies (**Table 1**), nine interventions (64·28%) were delivered online or internet-based (15, 16, 29, 31, 32, 34–37), and five (35.17%) were delivered face-to-face (13, 14, 28, 30, 33). Six interventions (42·85%) were mainly self-care or without a trained specialist (15, 16, 29, 31, 34, 35), while the other eight (57·14%) were mainly provided by trained specialists (13, 14, 28, 30, 32, 33, 36, 37). The median number of sessions was 8 with an interquartile range from 3 to 10. The longest intervention duration was 18 weeks with a weekly session (33) and the shortest duration was 10 minutes with only one online video session (35). Based on the duration and session, there were five low-frequency interventions (35·17%) (16, 29, 31, 34, 35) with a duration of less than four weeks or a maximum of three sessions and eight high-frequency interventions (57·14%) (13–15, 30, 32, 33, 36, 37) with a duration of more than four weeks or a maximum of 18 sessions. All interventions have a basic standardized process. At the end of the intervention, the median adherence rate was 90% with IQR from 74% to 100%. Most studies had good adherence of more than 80%. Six studies (14, 15, 30–32, 37) had recorded adverse events during the intervention and four studies (14, 15, 31, 37) reported no adverse events. One study reported five active suicidalities (four in the intervention group and one in the comparison group) (30). Another study reported two suicidal ideation and/or attempts (one in the intervention group and one in the comparison group) (32). The rest had no adverse event information. Few studies reported adverse events, but adverse events were generally rare.

Cognitive behavior therapy (CBT) was one of the most widely adopted therapeutic interventions for the treatment of various mental health disorders. Seven interventions included in this review were developed based on the principles of CBT (13, 16, 28, 30, 32, 36, 37). Another four studies adopted psychoeducation as a main approach to improve the mental health of participants (14, 15, 34, 35). Two further studies adopted expressive writing to improve mental health (29, 31). Meanwhile, most interventions adopted more than one therapy or technique. Some interventions based on CBT also contain the contents of psychoeducation (37), mindfulness (13, 30), or dialectical behavior therapy (28). Psychoeducation might involve cognitive adjustment (34), or dialectical discussion as well (14). Most interventions were adapted for sexual minorities and mainly focused on minority stress (13, 15, 16, 30–32, 36, 37). The minority stress theory was commonly adopted in the intervention design, development, and implementation which believed that sexual minorities usually faced extensive distal and proximal stress leading to poor mental health (3). It provided a comprehensive framework to understand the stressors from different levels and aspects. Researchers can design and develop their interventions based on that making their interventions more targeted and specific. Several studies adopted this theory into CBT interventions to help participants identify stressors and develop strategies to enhance stress coping (13, 16, 30, 32, 36, 37). Some studies adopted this model in psychoeducation materials to strengthen the self-adjustment (15).

**Figure 2:**
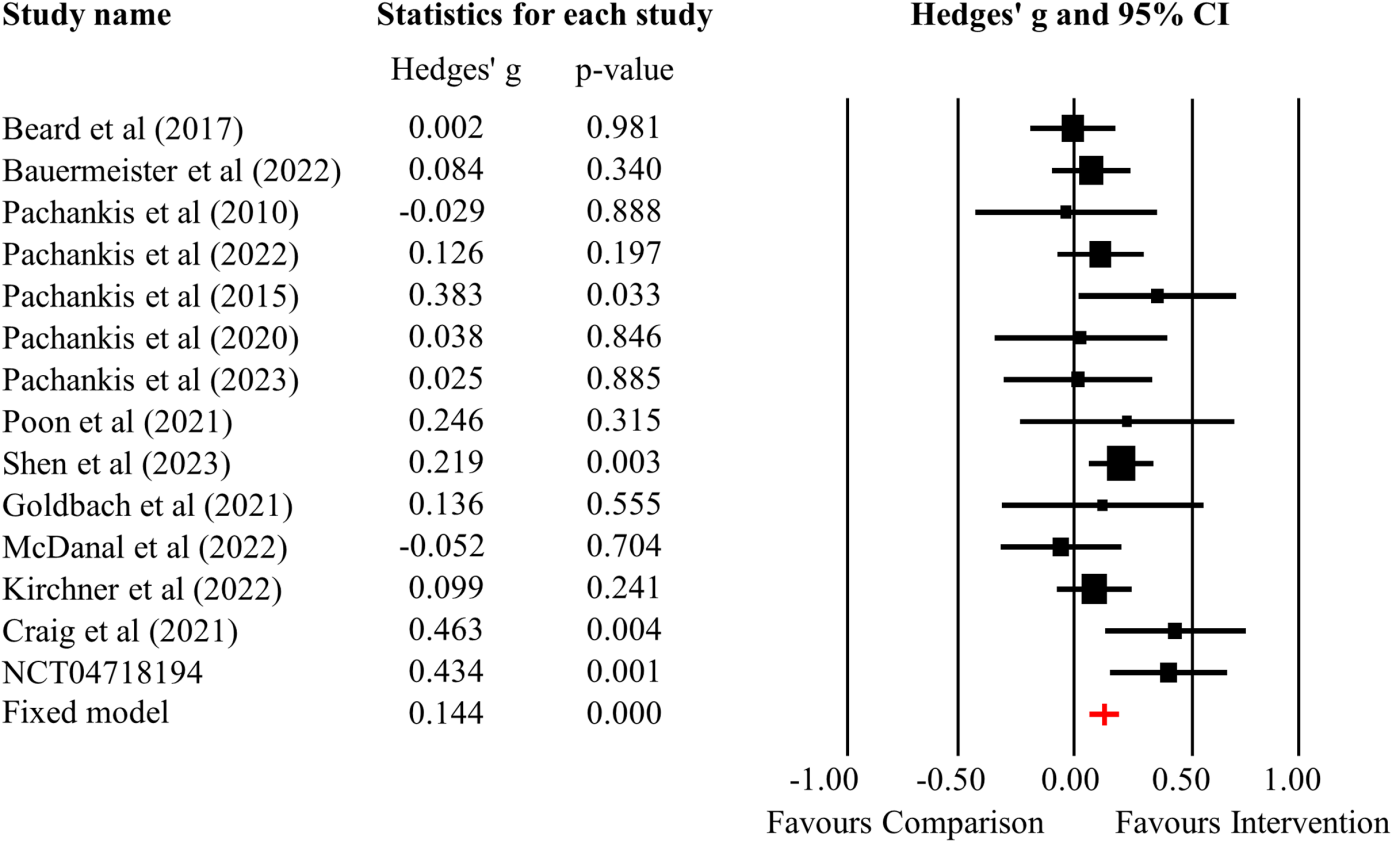
Forest plot of the overall effectiveness of psychosocial interventions on mental health outcomes.

The psychosocial intervention had a significant effect size on improving overall mental health status (Hedges’ *g* = 0·14, 95%CI: 0·08-0·21, *p*<0·001, n=14) (**Figure 2**). There was no significant heterogeneity between studies (*I^2^* = 28·23%, *Q* = 18·11, *p* = 0·15). Sensitivity analysis showed that effect size remained significant after excluding four studies considered to be at high risk-of-bias (Hedges’ *g* = 0·15, 95%CI: 0·07-0·22, *p*<0·001, *I^2^* = 9·51%, n=10). The overall effect size at the follow-up was 0·14 (95%CI: -0·05-0·33, *p* = 0·16, *I^2^* = 81·95%, n=8).

For specific outcomes at post-intervention (**Table 2**), the effect size was 0·25 (95%CI: 0·09-0·41, *p* = 0·002, *I^2^* = 57·77%, n=11) for depressive symptoms, 0·20 (95%CI: 0·12-0·29, *p*<0·001, *I^2^* = 46·78%, n=9) for anxiety symptoms, and 0·19 (95%CI: 0·10-0·28, *p*<0·001, *I^2^* = 11·49%, n=6) for substance abuse. The intervention had a positive effect on stress (Hedges’ *g* = 0·18, 95%CI: 0·03-0·33, *p* = 0·02, *I^2^* = 0%, n=5), coping (Hedges’ *g* = 0·21, 95%CI: 0·06-0·36, *p* = 0·006, *I^2^* = 0%, n=5), emotion (Hedges’ *g* = 0·16, 95%CI: 0·06-0·25, *p* = 0·001, *I^2^* = 45·86%, n=8), and identity (Hedges’ *g* = 0·19, 95%CI: 0·07-0·30, *p* = 0·001, *I^2^* = 49·60%, n=5). The intervention didn’t have a positive effect on suicidal ideation (Hedges’ *g* = 0·01, *p*>0·05) and social function (Hedges’ *g* = 0·01, *p*>0·05). At follow-up, the intervention groups had a superior effect on depressive symptoms (Hedges’ *g* = 0·36, 95%CI: 0·09-0·70, *p* = 0·044, *I^2^* = 86·14%, n=6), anxiety symptoms (Hedges’ *g* = 0·42, 95%CI: 0·01-0·82, *p* = 0·044, *I^2^* = 89·06%, n=5) and substance abuse (Hedges’ *g* = 0·23, 95%CI: 0·04-0·42, *p* = 0·015, *I^2^* = 50·07%, n=5).

**Table 2:** Effect size of specific outcomes.

Outcomes	Time point	Hedges’ g	Lower limit	Upper limit	P-value	I-square
Overall	Post intervention	0.144	0.080	0.207	<0.001	28.23%
Mental health						
Depression symptom	Post intervention	0.249	0.092	0.406	0.002	57.77%
Anxiety symptom	Post intervention	0.204	0.120	0.288	<0.001	46.78%
Substance abuse	Post intervention	0.190	0.099	0.280	<0.001	11.49%
Suicidal ideation	Post intervention	0.013	-0.186	0.212	0.898	0.00%
Psychosocial function						
Stress	Post intervention	0.178	0.028	0.328	0.020	0.00%
Coping	Post intervention	0.212	0.060	0.364	0.006	0.00%
Emotion	Post intervention	0.157	0.063	0.251	0.001	45.86%
Social function	Post intervention	0.013	-0.256	0.281	0.926	67.13%
Identity	Post intervention	0.188	0.073	0.303	0.001	49.60%
Overall	Follow-up	0.140	-0.054	0.333	0.158	81.95%
Mental health						
Depression symptom	Follow-up	0.356	0.009	0.703	0.044	86.14%
Anxiety symptom	Follow-up	0.417	0.012	0.822	0.044	89.06%
Substance abuse	Follow-up	0.231	0.044	0.418	0.015	50.07%
Suicidal ideation	Follow-up	0.027	-0.258	0.313	0.851	0.00%
Psychosocial function						
Stress	Follow-up	-0.009	-0.224	0.206	0.933	0.00%
Coping	Follow-up	0.609	-0.318	1.537	0.198	91.82%
Emotion	Follow-up	0.165	-0.162	0.491	0.323	87.04%
Social function	Follow-up	0.151	-0.347	0.649	0.553	78.41%
Identity	Follow-up	0.287	-0.017	0.591	0.064	74.57%

Online interventions had positive effects on the overall mental health status, coping, emotion, and identity, while face-to-face interventions had positive effects on depressive symptoms, anxiety symptoms, and substance abuse (**Table 3**). Interventions only for MSM and for LGBTQ+ both were effective in overall mental health status. Intervention for MSM had positive effects on depressive symptoms, anxiety symptoms, substance abuse, and identity, while intervention for LGBTQ+ had positive effects on stress and emotion. The self-care interventions had better effects on emotion and overall status, and the interventions provided by trained specialists had better effects on depressive symptoms, anxiety symptoms, substance abuse, stress, coping, and overall status. The low-frequency interventions had better effects on emotion and overall status, and the high-frequency interventions had better effects on depressive symptoms, anxiety symptoms, substance abuse, stress, coping, social function, and overall status. However, these variables had no moderating effect except for the effect sizes of low- and high-frequency interventions on social functioning (*p* = 0·001). We assessed the moderating effects of “age” and “number of sessions” by meta-regression, but there was no significant effect. Based on the above research findings, we can select appropriate intervention methods in practice by considering the specific context. When mental health resources are limited, short-term online self-care interventions may be a more suitable option.

**Table 3:** Subgroup analysis of effect sizes.

Outcomes	Online intervention		Face-to-face intervention		P-value between group
	Hedges’ g (95% CI)	P-value	Hedges’ g (95% CI)	P-value	
Overall	0.15 (0.05 to 0.26)	0.005	0.12 (-0.003 to 0.23)	0.055	0.640
Mental health					
Depression symptom	0.26 (-0.03 to 0.55)	0.074	0.23 (0.06 to 0.41)	0.009	0.855
Anxiety symptom	0.25 (-0.10 to 0.60)	0.162	0.19 (0.10 to 0.29)	<0.001	0.762
Substance abuse	0.03 (-0.20 to 0.25)	0.825	0.23 (0.08 to 0.37)	0.002	0.141
Suicidal ideation	-0.10 (-0.57 to 0.37)	0.673	0.04 (-0.18 to 0.26)	0.734	0.598
Psychosocial function					
Stress	0.26 (-0.05 to 0.56)	0.096	0.11 (-0.13 to 0.36)	0.356	0.470
Coping	0.22 (0.01 to 0.44)	0.044	0.30 (-0.08 to 0.68)	0.122	0.723
Emotion	0.18 (0.02 to 0.35)	0.032	0.01 (-0.38 to 0.40)	0.960	0.425
Social function	-0.06 (-0.55 to 0.44)	0.823	0.03 (-0.27 to 0.33)	0.828	0.767
Identity	0.23 (0.01 to 0.46)	0.041	0.16 (-0.30 to 0.63)	0.496	0.782
Outcomes	MSM-only intervention		LGBT+ intervention		P-value between group
	Hedges’ g (95% CI)	P-value	Hedges’ g (95% CI)	P-value	
Overall	0.24 (0.04 to 0.44)	0.019	0.12 (0.04 to 0.20)	0.004	0.272
Mental health					
Depression symptom	0.40 (0.09 to 0.71)	0.013	0.14 (-0.02 to 0.29)	0.086	0.142
Anxiety symptom	0.49 (0.07 to 0.92)	0.024	0.10 (-0.04 to 0.24)	0.157	0.088
Substance abuse	0.23 (0.04 to 0.42)	0.017	0.08 (-0.11 to 0.26)	0.410	0.253
Suicidal ideation	NA	NA	0.01 (-0.19 to 0.21)	0.898	NA
Psychosocial function					
Stress	0.10 (-0.17 to 0.37)	0.477	0.22 (0.03 to 0.40)	0.023	0.484
Coping	0.28 (-0.02 to 0.57)	0.066	0.22 (-0.02 to 0.47)	0.076	0.783
Emotion	0.19 (-0.36 to 0.73)	0.499	0.14 (0.04 to 0.24)	0.007	0.867
Social function	-0.01 (-0.65 to 0.63)	0.978	-0.01 (-0.17 to 0.16)	0.948	0.992
Identity	0.37 (0.07 to 0.68)	0.017	0.12 (-0.11 to 0.34)	0.304	0.189
Outcomes	Self-care intervention		Intervention with specialists		P-value between group
	Hedges’ g (95% CI)	P-value	Hedges’ g (95% CI)	P-value	
Overall	0.11 (0.03 to 0.20)	0.008	0.20 (0.07 to 0.35)	0.003	0.240
Mental health					
Depression symptom	0.11 (-0.09 to 0.31)	0.286	0.30 (0.10 to 0.50)	0.003	0.184
Anxiety symptom	0.09 (-0.12 to 0.30)	0.406	0.28 (0.10 to 0.47)	0.003	0.180
Substance abuse	-0.10 (-0.55 to 0.34)	0.651	0.20 (0.11 to 0.30)	<0.001	0.188
Suicidal ideation	NA	NA	0.01 (-0.19 to 0.21)	0.898	NA
Psychosocial function					
Stress	0.14 (-0.09 to 0.36)	0.231	0.22 (0.01 to 0.42)	0.042	0.608
Coping	0.09 (-0.13 to 0.31)	0.415	0.33 (0.12 to 0.55)	0.002	0.119
Emotion	0.12 (0.00 to 0.23)	0.050	0.28 (-0.06 to 0.63)	0.108	0.366
Social function	-0.30 (-1.07 to 0.47)	0.445	0.15 (-0.15 to 0.45)	0.321	0.283
Identity	0.23 (-0.06 to 0.52)	0.126	0.26 (-0.02 to 0.53)	0.066	0.888
Outcomes	Low-frequency intervention		High- frequency intervention		P-value between group
	Hedges’ g (95% CI)	P-value	Hedges’ g (95% CI)	P-value	
Overall	0.12 (0.02 to 0.22)	0.017	0.22 (0.10 to 0.34)	<0.001	0.212
Mental health					
Depression symptom	0.14 (-0.35 to 0.64)	0.574	0.31 (0.12 to 0.50)	<0.001	0.538
Anxiety symptom	-0.02(-0.50 to 0.45)	0.928	0.29 (0.11 to 0.47)	0.002	0.225
Substance abuse	-0.10 (-0.55 to 0.34)	0.651	0.21 (0.08 to 0.35)	0.002	0.182
Suicidal ideation	-0.10 (-0.57 to 0.37)	0.673	0.02 (-0.57 to 0.62)	0.936	0.745
Psychosocial function					
Stress	NA	NA	0.18 (0.03 to 0.33)	0.020	NA
Coping	NA	NA	0.21 (0.06 to 0.36)	0.006	NA
Emotion	0.12 (0.00 to 0.23)	0.050	0.28 (-0.06 to 0.63)	0.11	0.366
Social function	-0.74 (-1.29 to -0.18)	0.009	0.17 (-0.03 to 0.37)	0.090	0.002
Identity	0.43 (-0.10 to 0.95)	0.109	0.11 (-0.09 to 0.31)	0.284	0.267

**Figure 3:**
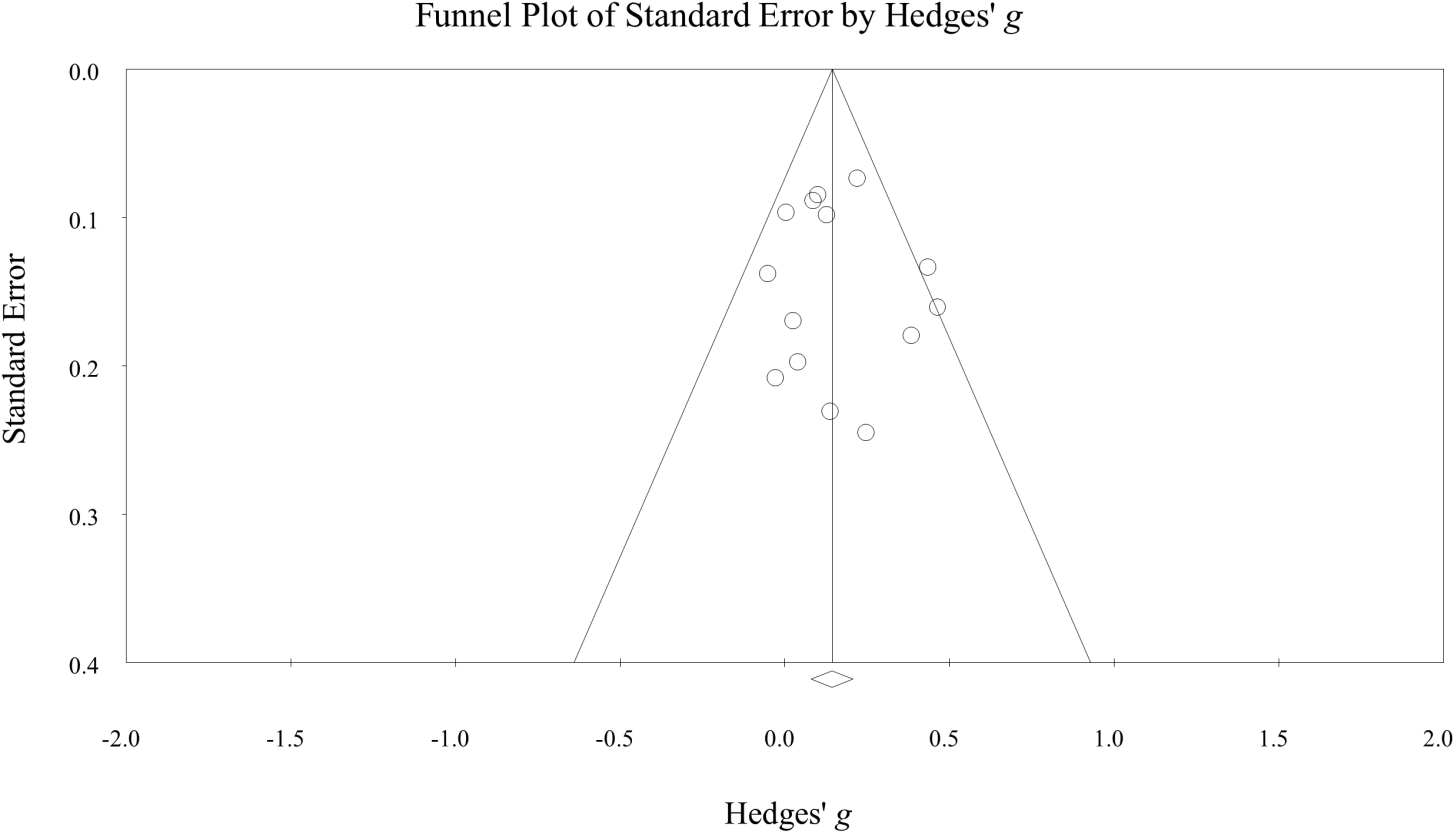
Funnel plot.

Three RCT studies and one quasi-experimental study were assessed at “high risk-of-bias” or had “serious bias”. Seven studies were assessed as having “some concerns” or having “moderate bias”, and four studies were at “low risk-of-bias” (S8-9). The funnel plot (**Figure 3**) and Egger’s test (intercept: 0·32, *p* = 0·72) did not show a significant publication bias. The quality of evidence was estimated by GRADE to be moderate (S10). The certainty was downgraded from high to moderate because of studies with a high risk-of-bias. This downgrade reflects how bias may influence the reliability of the results. However, the overall findings still support the effectiveness of psychosocial interventions. By downgrading the rating, we aim to provide a more cautious interpretation and highlight the need for future research with more rigorously designed studies to strengthen the evidence base.

## 4 Discussion

This systematic review and meta-analysis provide a comprehensive evaluation of the current psychosocial interventions for MSM mental health. An overall benefit of psychosocial intervention for mental health in MSM was observed in this review with a pooled effect size of 0·14 (95%CI: 0·08-0·21), which supported the research hypothesis. The results showed that the psychosocial interventions had more improvements in general mental status, mental health, and psychosocial functions than the comparison groups. No publication bias or substantial heterogeneity was found in this study. Moreover, the subgroup analyses based on the characteristics of interventions would provide additional information for future intervention development. However, the effectiveness of these interventions must be considered in the context of various external factors that drive poor mental health outcomes for MSM. Violence (38, 39), stigma (40), and discrimination (41) are key contributors that exacerbate mental health disparities. These social stressors not only negatively impact psychological well-being but also limit MSM’s access to mental health support and resources. In addition to providing interventions, addressing these root causes of poor mental health is crucial for improving outcomes in this population. Based on our findings, researchers could have a better understanding of psychosocial interventions for MSM and their characteristics and further develop more effective interventions.

The result of this study indicated that the interventions had a small effect size on the overall mental health status of MSM, which was in line with another study conducted by Pantalone et al. They reported a similar effect size (0·20) of intervention for mental health and substance abuse in MSM (18). For the depression symptoms, our study found a small-to moderate effect (0·26) of intervention, similar to a study for MSM living with HIV (0·28) (12). We found a small effect of intervention for anxiety symptoms (0·21) and stress symptoms (0·18), consistent with another study for sexual and gender minorities (17). From these studies, it was indicated that psychosocial interventions tend to have a small-to-moderate impact on the mental health of MSM (42). Apart from these symptoms, we also assessed the effectiveness in coping, identity, social function, and emotion aspects providing a more comprehensive assessment in this study and finding positive effects in intervention groups. Even if the effect is small, when applied to a large population, it may lead to meaningful improvements at the population level. Furthermore, if interventions are affordable and accessible, their widespread implementation could yield significant public health benefits, especially for communities with limited access to mental health resources.

No significant moderating effect was found for subgroup analyses. While no significant differences were found between online interventions and face-to-face interventions, the online intervention appeared to be more consistent in improving psychosocial aspects (e.g., coping, emotion, and identity), whereas the face-to-face intervention appeared to be more consistent in improving clinical symptoms (e.g., depression, anxiety, and substance abuse). MSM-only interventions were effective on overall status, mental health aspects, and identity, while LGBTQ+ interventions were effective on overall status and psychosocial aspects. However, there were no significant differences between the two interventions. Both self-care interventions and interventions provided by specialists had the same effect on overall mental health. The same results were also shown in the low and high-frequency interventions. This similarity may be attributed to the high degree of overlap between interventions. Most self-care interventions tend to be brief and low-frequency, whereas most high-frequency interventions tend to be provided by specialists. We only found significant moderating effects on social function between low- and high-frequency interventions. It might be that there were fewer studies measuring social function and therefore our results are susceptible to the extremes of particular studies. Although overall statistical significance was not achieved, the trends and consistency observed in the data showed that interventions with certain characteristics may have potential advantages in specific areas, which may still be important and have a positive impact on the development of future interventions. In high-income countries, psychosocial interventions benefited from strong health systems, sufficient trained providers, and supportive policies, making care more accessible for MSM. In contrast, in low- and middle-income countries (LMICs), limited resources made community-based or online approaches more scalable and acceptable. Self-care and digital delivery overcame barriers of distance and workforce shortages, while in urban settings, more intensive face-to-face interventions by professionals were feasible due to greater service availability.

In terms of intervention therapies, more than half of the studies adopted the principle of CBT. CBT is generally considered to be more standardized, structured, and focused as compared to other forms of psychotherapy, which makes it easier for trained intervention providers to implement (43). Moreover, CBT is usually a short-term intervention that can be delivered in various formats such as online, individual or group-based sessions (44). The core approach was to identify negative thought patterns and replace them with more positive, realistic ones (45). This process helped individuals to develop practical skills for coping with difficult emotions and situations (46). The CBT interventions included in this study have shown a positive impact on improving mental health by enhancing emotion regulation, cognitive reconstruction, strengthening coping mechanisms for minority stigma and stress, and facilitating participants to develop motivation and skills for behavioral change.

Other commonly adopted interventions were psychoeducation and expressive writing. Psychoeducation is a cost-effective and efficient way to improve mental health and can be conducted quickly with large sample sizes (47, 48). These interventions were usually passive education by providing information on emotion regulation, sexual minority communities, guidance on stress coping, or dealing with the stress of coming out to participants (15). Information is usually provided through web pages or online videos so that participants can access these resources at a flexible time (14, 15). Psychoeducation for MSM or LGBTQ+ was usually in the form of brief, short-term, or single-session intervention (35). The adoption of social media and Internet technology facelifted psychoeducation, making it easier to disseminate, more effective, and more attractive. Expressive writing prompts participants to write about their sexual minority-related stressful events, which can facilitate cognitive processing of unresolved psychological stressors (28, 31). For MSM, writing about their stressful experiences may enhance exposure to stress-related cues and eventually habituate them. Participants can benefit from exposure and cognitive adjustment during writing tasks.

### 4.1 Strengths

Several strengths of this study should be emphasized. First, we adopted a comprehensive perspective to evaluate the overall effectiveness of mental health interventions. Our results include not only common clinical symptoms (like depression and anxiety) but also easily overlooked psychosocial functions (like identity, coping, and social function), as well as calculating an overall effect size. We provide extensive evaluations from the overall to the specific. Second, the detailed subgroup analysis based on the study characteristics reflects the impact of different intervention settings on outcomes. It also provided information and implications for further intervention. Third, most of the studies were published within the last 5 years, therefore the present study largely reflects a recent trend and the current state of research in MSM mental health interventions.

### 4.2 Limitations

This study has some limitations. First, the inclusion of interventions for the LGBTQ+ population might increase the heterogeneity of this study. While the content of the two interventions (MSM intervention and LGBTQ+ interventions) is generally the same, the populations receiving LGBTQ+ interventions are much more diverse, and the same intervention may have different effects on different populations. Second, the outcomes were assessed by self-reported scales which could be subjective. This is mainly limited by the fact that self-rated scales are more efficient in measuring psychological aspects compared to clinician-rated scales. Nevertheless, there still might be measurement bias using self-reported scales as participants might exaggerate the effects of the intervention, or the actual effects might be vulnerable to the subjective state of the participant at the time of measurement. The third limitation is the variability in the definitions and measurement of broad concepts such as “emotion,” “identity,” and “coping.” Different scales and definitions were used to assess these outcomes, which may have led to inconsistencies in the results. The lack of standardized definitions for these constructions could impact the comparability of findings and contribute to variability in the reported effects. Fourth, limited to the characteristics of including studies, the results should be interpreted with more discretion. The participants of the included studies were mostly teenagers and young adults thus the results might not be able to draw conclusions about middle-aged or senior participants. Fifth, although our study reported some statistically significant results, statistical significance does not often equal clinical significance. The overall effect size was small. From a conservative perspective, further research is needed to see if such an effect size can represent a true clinical improvement.

### 4.3 Implications

This study would provide some clinical and policy implications. In the *Comprehensive mental health action plan 2013–2030* (49), WHO recommended focusing on populations at high risk for mental illness, including the LGBTQ+ groups, and developing proactive strategies for them. MSM or other sexual and gender minorities are still having poor access to mental health services. Psychosocial interventions for MSM were effective approaches in reducing mental conditions and improving psychosocial functions. Even a brief single-session intervention might be helpful. Mental health service providers would develop the most optimal interventions based on practical needs and specific contexts, such as online self-care intervention for low-resource contexts, brief or low-frequency interventions for mild conditions and daily situations, specialist-delivered interventions for moderate to severe conditions. In addition, MSM and other social minorities face unique risk factors, discrimination, stigma, inequality, and sexual minority pressures. Their needs for mental health services are different from the general population, not only to alleviate clinical symptoms, but more importantly to reduce the minority stress, including building resilience, strengthening daily stress coping, regulating negative emotions, increasing self-exploration, and self-identification and accessing more social support and information. This study also found that current research mainly focuses on younger populations, with a lack of interventions targeting older MSM populations or non-Western countries and cultures. Future research should aim to diversify the populations studied.

Policy efforts should focus on reducing inequities in access to mental health care services for MSM, including promoting mental health knowledge and LGBTQ+ friendly awareness among health care providers, reducing barriers to MSM access to mental health services, and addressing the social determinants of mental health conditions. Developed countries like Netherlands and the United States has implemented mental health strategies for LGBTQ+ individuals (50), promoting inclusivity and reducing stigma. However, other countries still face barriers such as legal restrictions and discrimination (51). China has limited formal policy engagement, with community-based organizations providing informal support. Future research should assess the effectiveness of these policies and explore how they can be tailored to different contexts to ensure equitable access to mental health services and improve MSM well-being.

The clinicians and researchers would better adopt a comprehensive perspective to develop and provide mental health services to improve a wide range of mental health outcomes and overall well-being for MSM. More efforts should be made in practice and policy to increase access to adequate, high-quality and affordable mental health services for MSM and other minorities, reducing inequalities, promoting well-being, health equality and human rights. This systematic review and meta-analysis provided evidence for the effectiveness of psychosocial interventions for mental health and psychosocial functions in MSM. The optimal interventions for different psychological conditions of MSM and their effects should be explored in future studies. More interventional studies and strategies for MSM are needed to promote and improve the mental health of MSM and other vulnerable populations.

## 5 Conclusion

This systematic review and meta-analysis demonstrate the overall benefits of psychosocial interventions for MSM mental health, with a small-to-moderate effect size. These interventions could reduce clinical symptoms like depression, anxiety, and substance abuse while improving psychosocial functions such as self-identity, coping, and social function. Future research should explore the effectiveness of interventions across different socio-cultural contexts and evaluate the impact of policy measures, while also focusing on enhancing mental health accessibility, reducing minority stress, and promoting equity in MSM populations.

## Data Availability

The raw data supporting the conclusions of this article will be made available by the authors, without undue reservation.

## Footnotes

1
^‡^ IQR, Interquartile range.
