# Supplementary material for: Effectiveness of psychosocial interventions to improve the mental health in men who have sex with men: a systematic review and meta-analysis
Source: Front Psychiatry. 2025 Sep 22;16:1612755. doi: 10.3389/fpsyt.2025.1612755 (PMC12497705; doi:10.3389/fpsyt.2025.1612755)
Supplement: Supplementary file 1 [file Table1.docx]

Supplementary appendix

Table of Contents

[S1 Appendix: PRISMA 2020 checklist 2](#_Toc173922318)

[S2 Appendix: Search strategy for PubMed 5](#_Toc173922319)

[S3 Appendix: Search strategy for PsycINFO 6](#_Toc173922320)

[S4 Appendix: Search strategy for EmBase 7](#_Toc173922321)

[S5 Appendix: Search strategy for Cochrane 8](#_Toc173922322)

[S6 Appendix: Search strategy for Clinicaltrials.gov 9](#_Toc173922323)

[S7 Appendix: Search strategy for International Clinical Trials Registry Platform (ICTRP) 10](#_Toc173922324)

[S8 Appendix: Assessment of The Cochrane risk-of-bias tool for randomized trials (RoB-2) 11](#_Toc173922325)

[S9 Appendix: Assessment of The Risk of Bias in Non-randomized Studies–of Interventions (ROBINS-I) 12](#_Toc173922326)

[S10 Appendix: Assessment of Grading of Recommendations, Assessment, Development and Evaluations (GRADE) 13](#_Toc173922327)

# **S1 Appendix: PRISMA 2020 checklist**

| **Section and Topic** | **Item #** | **Checklist item** | **Location where item is reported** |
| --- | --- | --- | --- |
| **TITLE** | | |  |
| Title | 1 | Identify the report as a systematic review. | Title |
| **ABSTRACT** | | |  |
| Abstract | 2 | See the PRISMA 2020 for Abstracts checklist. | Abstract |
| **INTRODUCTION** | | |  |
| Rationale | 3 | Describe the rationale for the review in the context of existing knowledge. | Paragraph 1-4 |
| Objectives | 4 | Provide an explicit statement of the objective(s) or question(s) the review addresses. | Paragraph 5 |
| **METHODS** | | |  |
| Eligibility criteria | 5 | Specify the inclusion and exclusion criteria for the review and how studies were grouped for the syntheses. | Paragraph 6 |
| Information sources | 6 | Specify all databases, registers, websites, organisations, reference lists and other sources searched or consulted to identify studies. Specify the date when each source was last searched or consulted. | Paragraph 7 |
| Search strategy | 7 | Present the full search strategies for all databases, registers and websites, including any filters and limits used. | Paragraph 7 |
| Selection process | 8 | Specify the methods used to decide whether a study met the inclusion criteria of the review, including how many reviewers screened each record and each report retrieved, whether they worked independently, and if applicable, details of automation tools used in the process. | Paragraph 7 |
| Data collection process | 9 | Specify the methods used to collect data from reports, including how many reviewers collected data from each report, whether they worked independently, any processes for obtaining or confirming data from study investigators, and if applicable, details of automation tools used in the process. | Paragraph 9-10 |
| Data items | 10a | List and define all outcomes for which data were sought. Specify whether all results that were compatible with each outcome domain in each study were sought (e.g. for all measures, time points, analyses), and if not, the methods used to decide which results to collect. | Paragraph 9 |
|  | 10b | List and define all other variables for which data were sought (e.g. participant and intervention characteristics, funding sources). Describe any assumptions made about any missing or unclear information. | Paragraph 9 |
| Study risk of bias assessment | 11 | Specify the methods used to assess risk of bias in the included studies, including details of the tool(s) used, how many reviewers assessed each study and whether they worked independently, and if applicable, details of automation tools used in the process. | Paragraph 10 |
| Effect measures | 12 | Specify for each outcome the effect measure(s) (e.g. risk ratio, mean difference) used in the synthesis or presentation of results. | Paragraph 11-13 |
| Synthesis methods | 13a | Describe the processes used to decide which studies were eligible for each synthesis (e.g. tabulating the study intervention characteristics and comparing against the planned groups for each synthesis (item #5)). | Paragraph 11-13 |
|  | 13b | Describe any methods required to prepare the data for presentation or synthesis, such as handling of missing summary statistics, or data conversions. | Paragraph 11-13 |
|  | 13c | Describe any methods used to tabulate or visually display results of individual studies and syntheses. | Paragraph 11-13 |
|  | 13d | Describe any methods used to synthesize results and provide a rationale for the choice(s). If meta-analysis was performed, describe the model(s), method(s) to identify the presence and extent of statistical heterogeneity, and software package(s) used. | Paragraph 11-13 |
|  | 13e | Describe any methods used to explore possible causes of heterogeneity among study results (e.g. subgroup analysis, meta-regression). | Paragraph 13 |
|  | 13f | Describe any sensitivity analyses conducted to assess robustness of the synthesized results. | Paragraph 13 |
| **Reporting bias assessment** | 14 | Describe any methods used to assess risk of bias due to missing results in a synthesis (arising from reporting biases). | Paragraph 10 |
| Certainty assessment | 15 | Describe any methods used to assess certainty (or confidence) in the body of evidence for an outcome. | Paragraph 10 |
| **RESULTS** | | |  |
| Study selection | 16a | Describe the results of the search and selection process, from the number of records identified in the search to the number of studies included in the review, ideally using a flow diagram. | Paragraph 14 |
|  | 16b | Cite studies that might appear to meet the inclusion criteria, but which were excluded, and explain why they were excluded. | Paragraph 14 |
| Study characteristics | 17 | Cite each included study and present its characteristics. | Paragraph 15 |
| Risk of bias in studies | 18 | Present assessments of risk of bias for each included study. | Paragraph 22 |
| Results of individual studies | 19 | For all outcomes, present, for each study: (a) summary statistics for each group (where appropriate) and (b) an effect estimate and its precision (e.g. confidence/credible interval), ideally using structured tables or plots. | Paragraph 19 |
| Results of syntheses | 20a | For each synthesis, briefly summarise the characteristics and risk of bias among contributing studies. | Paragraph 16-21 |
|  | 20b | Present results of all statistical syntheses conducted. If meta-analysis was done, present for each the summary estimate and its precision (e.g. confidence/credible interval) and measures of statistical heterogeneity. If comparing groups, describe the direction of the effect. | Paragraph 19-21 |
|  | 20c | Present results of all investigations of possible causes of heterogeneity among study results. | Paragraph 16-18 |
|  | 20d | Present results of all sensitivity analyses conducted to assess the robustness of the synthesized results. | Paragraph 20-22 |
| Reporting biases | 21 | Present assessments of risk of bias due to missing results (arising from reporting biases) for each synthesis assessed. | Paragraph 22 |
| Certainty of evidence | 22 | Present assessments of certainty (or confidence) in the body of evidence for each outcome assessed. | Paragraph 22 |
| **DISCUSSION** | | |  |
| Discussion | 23a | Provide a general interpretation of the results in the context of other evidence. | Paragraph 23-25 |
|  | 23b | Discuss any limitations of the evidence included in the review. | Paragraph 29 |
|  | 23c | Discuss any limitations of the review processes used. | Paragraph 29 |
|  | 23d | Discuss implications of the results for practice, policy, and future research. | Paragraph 30-32 |
| **OTHER INFORMATION** | | |  |
| Registration and protocol | 24a | Provide registration information for the review, including register name and registration number, or state that the review was not registered. | Paragraph 8 |
|  | 24b | Indicate where the review protocol can be accessed, or state that a protocol was not prepared. | Paragraph 8 |
|  | 24c | Describe and explain any amendments to information provided at registration or in the protocol. | NA |
| Support | 25 | Describe sources of financial or non-financial support for the review, and the role of the funders or sponsors in the review. |  |
| Competing interests | 26 | Declare any competing interests of review authors. |  |
| Availability of data, code and other materials | 27 | Report which of the following are publicly available and where they can be found: template data collection forms; data extracted from included studies; data used for all analyses; analytic code; any other materials used in the review. |  |

# **S2 Appendix: Search strategy for PubMed**

(("Mental Health"[Mesh]) OR ("Mental Disorders"[Mesh]) OR ("Psychological Well-Being"[Mesh]) OR ("Suicide"[Mesh]) OR ("Stress, Psychological"[Mesh]) OR ("Social Stigma"[Mesh]) OR "stigma" OR "self-acceptance") OR ("Emotions"[Mesh]) **AND** (("Psychosocial Intervention"[Mesh]) OR ("Psychotherapy"[Mesh]) OR ("Behavioral Sciences"[Mesh]) OR ("Mental Health Services"[Mesh]) OR "intervention" OR "treatment" OR "training" OR "therapy" OR "program" OR "implement") **AND** ("men who have sex with men" OR "gay" OR "homosexual" OR ("Homosexuality"[Mesh]) OR ("Sexual and Gender Minorities"[Mesh]) OR "bisexual" OR ("Bisexuality"[Mesh]) OR "same-sex")

# **S3 Appendix: Search strategy for PsycINFO**

(MESH(mental health) OR MESH(mental disorder) OR MESH(psychological well-being) OR MESH(suicide) OR MESH(stress, psychological) OR MESH(social stigma) OR SUBJECT(stigma) OR SUBJECT(self-acceptance) OR MESH(emotion)) **AND** (MESH(psychosocial intervention) OR MESH(psychotherapy) OR MESH(behavioral sciences) OR MESH(mental health services) OR SUBJECT(intervention) OR SUBJECT(treatment) OR SUBJECT(training) OR SUBJECT(therapy) OR SUBJECT(program) OR SUBJECT(implement)) **AND** (SUBJECT(men who have sex with men) OR SUBJECT(gay) OR SUBJECT(homosexual) OR MESH(Homosexuality) OR MESH(sexual and gender minorities) OR SUBJECT(bisexual) OR MESH(Bisexuality) OR SUBJECT(same-sex))

# **S4 Appendix: Search strategy for EmBase**

Embase Classic+Embase

1 mental health/ 227422

2 mental disease/ 315501

3 psychological aspect/ 491822

4 psychological well-being/ 32892

5 suicide/ 72744

6 mental stress/ 104960

7 stigma/ 20115

8 social stigma/ 15897

9 self-acceptance.mp. 1346

10 emotion/ 140325

11 1 or 2 or 3 or 4 or 5 or 6 or 7 or 8 or 9 or 10 1251721

12 psychosocial intervention/ 2438

13 psychotherapy/ 112987

14 behavioral science/ 9638

15 mental health service/ 70926

16 intervention.mp. 1345634

17 treatment.mp. 9139075

18 training/ 122410

19 therapy/ 1412249

20 program.mp. 1145754

21 implement.mp. 138476

22 12 or 13 or 14 or 15 or 16 or 17 or 18 or 19 or 20 or 21 11814793

23 men who have sex with men/ 16196

24 homosexuality/ 23443

25 gay.mp. 17387

26 homosexual.mp. 20286

27 bisexual.mp. 14554

28 bisexuality/ 8714

29 same-sex.mp. 9899

30 "sexual and gender minority"/ 8837

31 23 or 24 or 25 or 26 or 27 or 28 or 29 or 30 73889

32 11 and 22 and 31 2903

# **S5 Appendix: Search strategy for Cochrane**

ID Search Hits

#1 MeSH descriptor: [Mental Health] explode all trees 3217

#2 MeSH descriptor: [Mental Disorders] explode all trees 105646

#3 MeSH descriptor: [Psychological Well-Being] explode all trees 34

#4 MeSH descriptor: [Suicide] explode all trees 2115

#5 MeSH descriptor: [Stress, Psychological] explode all trees 8850

#6 MeSH descriptor: [Social Stigma] explode all trees 635

#7 stigma 3825

#8 self-acceptance 149

#9 MeSH descriptor: [Emotions] explode all trees 39346

#10 #1 OR #2 OR #3 OR #4 OR #5 OR #6 OR #7 OR #8 OR #9 139502

#11 MeSH descriptor: [Psychosocial Intervention] explode all trees 267

#12 MeSH descriptor: [Psychotherapy] explode all trees 35196

#13 MeSH descriptor: [Behavioral Sciences] explode all trees 4392

#14 MeSH descriptor: [Mental Health Services] explode all trees 9439

#15 intervention 545918

#16 treatment 969140

#17 training 134890

#18 therapy 888558

#19 program 153610

#20 implement 10671

#21 #11 #12 OR #13 #14 #15 OR #16 OR #17 OR #18 OR #19 OR #20 1371373

#22 MeSH descriptor: [Sexual and Gender Minorities] explode all trees 535

#23 men who have sex with men 5499

#24 gay 1210

#25 homosexual 352

#26 MeSH descriptor: [Homosexuality] explode all trees 913

#27 bisexual 449

#28 MeSH descriptor: [Bisexuality] explode all trees 96

#29 same-sex 335

#30 #22 OR #23 OR #24 OR #25 OR #26 OR #27 OR #28 OR #29 7259

#31 #10 AND #21 AND #30 1011

# **S6 Appendix: Search strategy for Clinicaltrials.gov**

Condition or disease:

mental health OR mental disorder OR psychological well-being OR suicide OR suicidal OR trauma and stressor related disorders OR PTSD OR trauma OR substance-related disorders OR substance abuse OR stress OR stigma OR self-acceptance OR emotion

Other terms:

men who have sex with men OR gay OR homosexual OR homosexuality OR sexual and gender minorities OR bisexual OR Bisexuality OR same-sex OR LGB

Study type: interventional studies

Study Results: All studies

# **S7 Appendix: Search strategy for International Clinical Trials Registry Platform (ICTRP)**

In the Title:

men who have sex with men OR gay OR homosexual OR homosexuality OR sexual and gender minorities OR bisexual OR Bisexuality OR same-sex OR LGB

AND

In the Condition:

mental health OR mental disorder OR psychological well-being OR depressive disorder OR depression OR depressive OR depressed OR anxiety OR anxious OR suicide OR suicidal OR trauma and stressor related disorders OR PTSD OR trauma OR substance-related disorders OR substance abuse OR stress OR stigma OR self-acceptance OR emotion

Recruitment status is ALL

# **S8 Appendix: Assessment of The Cochrane risk-of-bias tool for randomized trials (RoB-2)**

| **Study** | **D1** | **D2** | **D3** | **D4** | **D5** | **Overall** |  |  |  |
| --- | --- | --- | --- | --- | --- | --- | --- | --- | --- |
| Bauermeister et al (2022) |  |  |  |  |  |  |  |  | Low risk |
| Pachankis et al (2010) |  |  |  |  |  |  |  |  | Some concerns |
| Pachankis et al (2022) |  |  |  |  |  |  |  |  | High risk |
| Pachankis et al (2015) |  |  |  |  |  |  |  |  |  |
| Pachankis et al (2020) |  |  |  |  |  |  |  | D1 | Randomisation process |
| Pachankis et al (2023) |  |  |  |  |  |  |  | D2 | Deviations from the intended interventions |
| Shen et al (2023) |  |  |  |  |  |  |  | D3 | Missing outcome data |
| Goldbach et al (2021) |  |  |  |  |  |  |  | D4 | Measurement of the outcome |
| Kirchner et al (2022) |  |  |  |  |  |  |  | D5 | Selection of the reported result |
| Craig et al (2021) |  |  |  |  |  |  |  |  |  |
| NCT04718194 |  |  |  |  |  |  |  |  |  |

# **S9 Appendix: Assessment of The Risk of Bias in Non-randomized Studies–of Interventions (ROBINS-I)**

| Study | Domain | Judgment |
| --- | --- | --- |
| Beard et al (2017) | Bias due to confounding | Moderate |
|  | Bias in selection of participants into the study | Serious |
|  | Bias in classification of interventions | Moderate |
|  | Bias due to deviations from intended interventions | Low |
|  | Bias due to missing data | Low |
|  | Bias in measurement of outcomes | Moderate |
|  | Bias in selection of the reported result | Low |
|  | Overall bias | Serious |
| Poon et al (2021) | Bias due to confounding | Moderate |
|  | Bias in selection of participants into the study | Moderate |
|  | Bias in classification of interventions | Moderate |
|  | Bias due to deviations from intended interventions | Low |
|  | Bias due to missing data | Low |
|  | Bias in measurement of outcomes | Moderate |
|  | Bias in selection of the reported result | Moderate |
|  | Overall bias | Moderate |
| McDanal et al (2022) | Bias due to confounding | Moderate |
|  | Bias in selection of participants into the study | Moderate |
|  | Bias in classification of interventions | Low |
|  | Bias due to deviations from intended interventions | Low |
|  | Bias due to missing data | Moderate |
|  | Bias in measurement of outcomes | Moderate |
|  | Bias in selection of the reported result | Moderate |
|  | Overall bias | Moderate |

# **S10 Appendix: Assessment of Grading of Recommendations, Assessment, Development and Evaluations (GRADE)**

**Date:** 2024-07-10
**Question:** Should psychosocial intervention vs comparison group be used for mental health and psychological function?
**Bibliography:**

| **Quality assessment** | | | | | | | **No of patients** | | **Effect** | | **Quality** | **Importance** |  |
| --- | --- | --- | --- | --- | --- | --- | --- | --- | --- | --- | --- | --- | --- |
|  |  |  |  |  |  |  |  |  |  |  |  |  |  |
| **No of studies** | **Design** | **Risk of bias** | **Inconsistency** | **Indirectness** | **Imprecision** | **Other considerations** | **Psychosocial intervention** | **Comparison group** | **Relative (95% CI)** | **Absolute** |  |  |  |
| **Overall mental health status (Better indicated by higher values)** | | | | | | | | | | | | |  |
| 14 | randomised trials | serious^1^ | no serious inconsistency | no serious indirectness | no serious imprecision | none | 1,209 | 1,467 | - | SMD 0.14 higher (0.08 to 0.21 higher) | ⊕⊕⊕O MODERATE | CRITICAL |  |
| **Depressive symptom (Better indicated by higher values)** | | | | | | | | | | | | |  |
| 11 | randomised trials | serious^2^ | serious^3^ | no serious indirectness | no serious imprecision | none | 623 | 848 | - | SMD 0.25 higher (0.09 to 0.41 higher) | ⊕⊕OO LOW | IMPORTANT |  |
| **Anxiety symptom (Better indicated by higher values)** | | | | | | | | | | | | |  |
| 9 | randomised trials | serious^4^ | no serious inconsistency | no serious indirectness | no serious imprecision | none | 550 | 773 | - | SMD 0.20 higher (0.12 to 0.29 higher) | ⊕⊕⊕O MODERATE | IMPORTANT |  |
| **Substance abuse (Better indicated by higher values)** | | | | | | | | | | | | |  |
| 6 | randomised trials | serious^4^ | no serious inconsistency | no serious indirectness | no serious imprecision | none | 372 | 596 | - | SMD 0.19 higher (0.1 to 0.28 higher) | ⊕⊕⊕O MODERATE | IMPORTANT |  |
| **Suicidal ideation (Better indicated by higher values)** | | | | | | | | | | | | |  |
| 3 | randomised trials | serious^5^ | no serious inconsistency | no serious indirectness | no serious imprecision | none | 147 | 412 | - | SMD 0.01 higher (0.19 lower to 0.21 higher) | ⊕⊕⊕O MODERATE | IMPORTANT |  |
| **Stress (Better indicated by higher values)** | | | | | | | | | | | | |  |
| 5 | randomised trials | serious^4^ | no serious inconsistency | no serious indirectness | no serious imprecision | none | 340 | 287 | - | SMD 0.18 higher (0.3 to 0.33 higher) | ⊕⊕⊕O MODERATE | IMPORTANT |  |
| **Coping (Better indicated by higher values)** | | | | | | | | | | | | |  |
| 5 | randomised trials | serious^5^ | no serious inconsistency | no serious indirectness | no serious imprecision | none | 289 | 299 | - | SMD 0.21 higher (0.06 to 0.34 higher) | ⊕⊕⊕O MODERATE | IMPORTANT |  |
| **Emotion (Better indicated by higher values)** | | | | | | | | | | | | |  |
| 8 | randomised trials | serious^2^ | no serious inconsistency | no serious indirectness | no serious imprecision | none | 767 | 808 | - | SMD 0.16 higher (0.06 to 0.25 higher) | ⊕⊕⊕O MODERATE | IMPORTANT |  |
| **Social function (Better indicated by higher values)** | | | | | | | | | | | | |  |
| 5 | randomised trials | serious^4^ | serious^3^ | no serious indirectness | no serious imprecision | none | 334 | 608 | - | SMD 0.01 higher (0.26 lower to 0.28 higher) | ⊕⊕OO LOW | IMPORTANT |  |
| **Identity (Better indicated by higher values)** | | | | | | | | | | | | |  |
| 5 | randomised trials | serious^6^ | no serious inconsistency | no serious indirectness | no serious imprecision | none | 516 | 527 | - | SMD 0.19 higher (0.07 to 0.30 higher) | ⊕⊕⊕O MODERATE | IMPORTANT |  |

^1^ Four studies were at low risk-of-bias, six studies had moderate concerns, and four studies were at high risk-of-bias.
^2^ Three studies were at high risk-of-bias.
^3^ I-square > 50%.
^4^ Two studies were at high risk-of-bias.
^5^ One study was at high risk-of-bias.
^6^ No explanation was provided.
